# Dynamic Mechanical Fatigue Behavior of Polymer Electrolyte Membranes for Fuel Cell Electric Vehicles Using a Gas Pressure-Loaded Blister

**DOI:** 10.3390/polym13234177

**Published:** 2021-11-29

**Authors:** Jun Hyun Lim, Jian Hou, Chang Hyun Lee

**Affiliations:** Department of Energy Engineering, Dankook University, Cheonan 31116, Korea; iyj2368@naver.com (J.H.L.); houjimmy@naver.com (J.H.)

**Keywords:** polymer electrolyte membrane, mechanical fatigue, durability, blister, fuel cell

## Abstract

This study reports on an innovative press-loaded blister hybrid system equipped with gas-chromatography (PBS-GC) that is designed to evaluate the mechanical fatigue of two representative types of commercial Nafion membranes under relevant PEMFC operating conditions (e.g., simultaneously controlling temperature and humidity). The influences of various applied pressures (50 kPa, 100 kPa, etc.) and blistering gas types (hydrogen, oxygen, etc.) on the mechanical resistance loss are systematically investigated. The results evidently indicate that hydrogen gas is a more effective blistering gas for inducing dynamic mechanical losses of PEM. The changes in proton conductivity are also measured before and after hydrogen gas pressure-loaded blistering. After performing the mechanical aging test, a decrease in proton conductivity was confirmed, which was also interpreted using small angle X-ray scattering (SAXS) analysis. Finally, an accelerated dynamic mechanical aging test is performed using the homemade PBS-GC system, where the hydrogen permeability rate increases significantly when the membrane is pressure-loaded blistering for 10 min, suggesting notable mechanical fatigue of the PEM. In summary, this PBS-GC system developed in-house clearly demonstrates its capability of screening and characterizing various membrane candidates in a relatively short period of time (<1.5 h at 50 kPa versus 200 h).

## 1. Introduction

Fuel cell electric vehicles (FCEVs) have recently attracted great attention as a powerful solution to minimize the generation of carbon dioxide as major exhaust gas from automotive vehicles. FCEVs are typically powered by polymer electrolyte membrane fuel cells (PEMFCs) that generate electrical energy from electrocatalytic hydrogen reactions [1]. It is well recognized that the polymer electrolyte membrane (PEM) is the key material for the development of PEMFC system which can ideally possess not only high electrochemical performances but also long lifetime. The PEM materials should easily carry the protons generated by the hydrogen oxidation reaction at the anode to the cathode, while also serving as a good barrier to prevent the mixing of hydrogen and oxygen gases, which should be supplied the anode and cathode sides separately [2,3]. Furthermore, an ideal PEM should have high mechanical toughness under various conditions (e.g., temperature, humidity, pressure, and/or chemical composition of fuel gases) where wide variations may occur during the operation of FCEVs. Otherwise, PEM materials may experience physical damages as pinholes, which may inevitably cause mechanical failure associated with undesirable gas penetration and electrochemical property deterioration [4].

To date, there have been diverse approaches to evaluate the real mechanical durability of the PEM [5,6,7]. One common approach is to examine the mechanical strength (e.g., tensile strength, yield stress, and percent elongation at yield and failure) of PEM after the exposure under a certain condition (e.g., 100% relative humidity at 80 °C) for a constant period [8,9,10,11,12]. This method can provide information on general mechanical properties of PEM in a static state, but in fact, the resulting values are still different from those obtained during dynamic FCEVs operations. Another approach, called as relative humidity (RH) cycling test, is to evaluate the changes of hydrogen crossover current density of PEM materials, which are assembled with electrodes in the membrane-electrode assembly (MEA) states exposed under repeated wet-dry cycles with a short interval (e.g., at 80 °C and ambient pressure between 0% RH for 2 min and 90 °C dew point for 2 min with airflow of 2 SLPM on both sides) [13,14]. It has been used as a representative method to determine the mechanical durability of PEM. This approach still has a critical technical issue: the measured mechanical durability can be highly affected by the MEA components (e.g., electrodes or their interfaces) more than PEM materials themselves. Moreover, it is not possible to evaluate the mechanical durability in a short period of time. Based on the literature reports, this method requires about 56 days to verify whether the mechanical durability of a PEM satisfies the 2020 target (e.g., 20,000 cycles) recommended by the U.S. Department of Energy [15]. For these reasons, it is necessary to develop a new method that can validate the mechanical durability of PEM materials in a short period of time under dynamic FCEVs operation conditions.

Recently, an alternative method has proposed by the air leak test using a homemade pressure-loaded blister system, which can apply biaxial air pressure stimuli to induce cyclic fatigue damage [16,17]. This dynamic mechanical evaluation can be carried out through the blistering test in a very short time (e.g., few minutes). Unfortunately, it has revealed several technical issues as the following: the air leak test employs dry air as a pressure-loaded gas which is somewhat different from real gases used during FCEVs operations. In contrast to static stationary applications in which there is no pressure difference (ΔP = 0) between anode and cathode, PEM materials in the FCEVs are subjected to the local pressure of feed gases (e.g., H_2_, O_2_, and air) acting as dynamic mechanical stimuli repeatedly applied in short intervals. Consequently, the influence of feed gas on the dynamic mechanical failure of PEM is highly dependent upon FCEVs operation modes. For example, the inadequate oxygen supply in a high current density region may cause a relatively high hydrogen pressure, which may result in fast hydrogen crossover through the PEM and thermal degradation on the electrode layers [18]. Another technical issue is whether the burst resistance of PEM is meaningful as a parameter to indirectly predict the membrane lifetime, because the reduction of electrochemical performances is caused by a small amount of permeated gas (e.g., H_2_) before a large amount of gas leakage occurring after PEM rupture.

In this study, we aimed to solve the limitations of the above-mentioned press-loaded blister approach. Our developed press-loaded blister hybrid system linked with gas chromatography (PBS-GC) can resolve several technical issues by using the real feeding gases used in FCEVs and controlling the temperature and relative humidity simultaneously during fuel cell operation. Here, a 15% loss of mechanical resistance by the PBS-GC system was chosen as the standard cut-off value instead of the PEM rupture [17]. The variation of this standard cut-off value at different kinds of feeding gas, temperature, and humidity conditions was further analyzed. Additionally, the changes of corresponding proton conductivity were evaluated after the pressure-loaded blistering. Lastly, the relationships between the dynamic mechanical aging time by the pressure-loaded blistering and the gas permeability rate was also studied. Designing and validating this type of system will allow for developing a simple and rapid test protocol for various membranes.

## 2. Materials and Methods

### 2.1. Materials

For all experiments, two kinds of commercially available PEMs (Chemours Company, Wilmington, DE, USA) were purchased and investigated in this study: Chemours’s the non-reinforced Nafion^TM^ 211 and the composite Nafion^TM^ XL membranes with a nominal thickness of 25 μm and 27 μm, respectively. These two membranes have similar chemical compositions based on perfluorosulfonic acid (PFSA) and the basic physical properties of them are shown in Table 1 [19,20].

### 2.2. Pressure-Loaded Blister Hybrid System Design and the PEMs Dynamic Mechanical Resistance Evaluation

**Figure 1:**
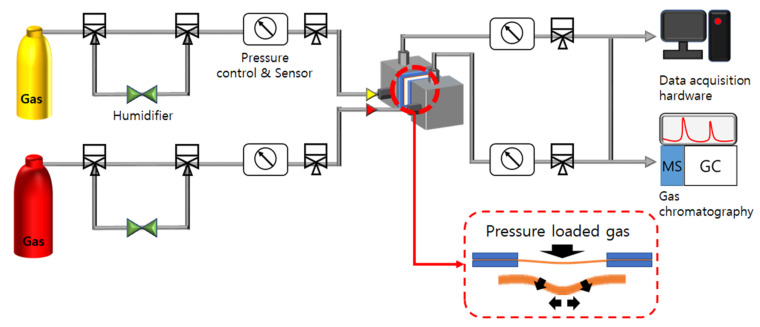
Homemade pressure-loaded blister hybrid system connected with GC.

We have designed a PBS-GC system that was then manufactured by the CNL Energy Co. Ltd. (Seoul, Korea) to simulate operating conditions similar to those applied on the PEM in FCEVs. The dynamic mechanical resistance on the PEM was then evaluated. Figure 1 depicts the concept of installing a dried circular membrane sample (diameter of 3 cm) using the PBS-GC system, then the membrane sample was loaded with a gas (e.g., H_2_, O_2_, or air) pressure on the anode side and a constant argon gas pressure on the cathode side, at four temperature and humidity combinations, two different temperatures (65 °C, 80 °C), and two different relative humidity (RH50%, RH100%) each. The gas loading pressure was controlled at 50 kPa, 100 kPa, and 200 kPa. At each test, the elapsed time of a 15% loss of mechanical resistance was recorded and chosen as the standard cut-off value.

### 2.3. Proton Conductivity & Nanostructure Changes after Pressure-Loaded Blistering under Specific Conditions

Proton conductivity is the primary characterization factor of the PEM, which can often be determined by the four-probe alternating current (ac) impedance spectroscopy method before and after pressure-loaded blistering [21]. All the membrane samples were analyzed in a hydrated state and placed in a constant temperature and humidity chamber. Final proton conductivity (σ, Scm^−1^) of each sample (dimension: 1 cm × 4 cm) was obtained by the following Equation (1): [21,22]

(1) $$\mathrm{Proton}\;\mathrm{conductivity}\;\sigma \;[S/{\mathrm{cm}}^{2}]=\frac{l}{R\times S}$$

where σ is proton conductivity, *R* is resistance, *l* is the distance between electrodes, and *S* is the cross-sectional area of the membrane.

Structural changes (the average distance changes between the hydrophilic domain of the PEM) before and after pressure-loaded blistering were examined by Small-angle X-ray scattering (SAXs, Model Bl 4C SAXs II). SAXS was done using X-ray beam with a wavelength of 0.07 nm, beam flux of 1 × 10^12^ ph/s, beam size of 100 (V) × 300 (H) μm^2^ and sample-to-detector distance of 1 m [23]. The distance of the hydrophilic domain (D, Å) was calculated using Bragg’s equation as in Equation (2) [24,25].

(2) $$D\;[Å]=\frac{2\pi }{q_{max}}$$

### 2.4. Hydrogen Permeability Changes after Pressure-Loaded Blistering

Hydrogen permeation is an unavoidable phenomenon in PEMFCs, which may lead to the formation of pinholes on the membrane and lower the electrochemical performance and durability [26]. Hereon, the gas permeability (P, cm^3^ cm cm^−2^ s^−1^) was determined by monitoring the gradient concentration through the membrane sample between a single cell constructed for the PBS-GC system and fed into hydrogen gas and argon gas under humidity 50% and temperature 80 °C conditions on each side of electrode. The hydrogen concentration was then detected using gas chromatography (Agilent Technologies, 490 Micro GC, CA, USA). The hydrogen permeability was calculated according to the following Equation (3): [11,27]

(3) $$P\;[{\mathrm{cm}}^{2}/s]=\frac{V_{B}\times l}{C_{A}\times A}\times \frac{C_{B}}{t}$$

where *V_B_* is the volume of the lower chamber in cm^3^, *l* is the membrane thickness in cm, *C_A_* and *C_B_* were concentrations in the feed gas and permeate, respectively. *A* is the exposed surface area of the membrane in cm^2^, *t* is the time.

## 3. Results

### 3.1. Evaluation of Mechanical Resistance on Specific Conditions

**Figure 2:**
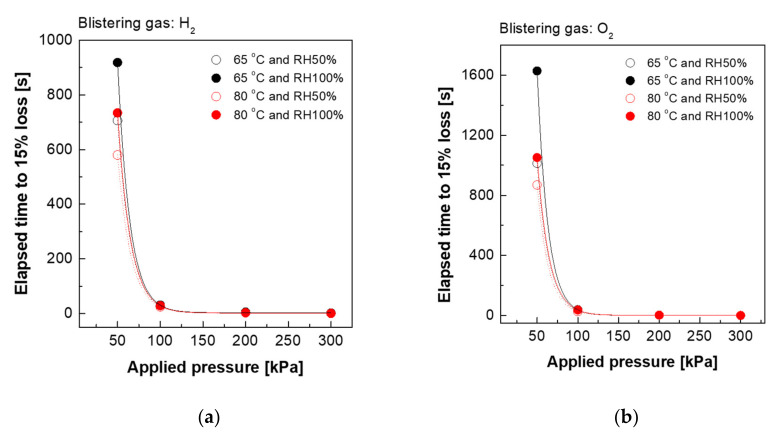
Evaluation of mechanical loss (**a**) blistering gas: hydrogen and (**b**) blistering gas: oxygen under various conditions (Nafion 211).

**Figure 3:**
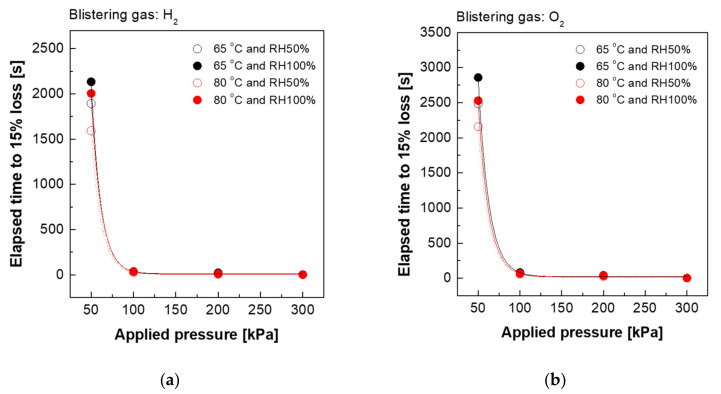
Evaluation of mechanical loss (**a**) blistering gas: hydrogen and (**b**) blistering gas: oxygen under various conditions (Nafion XL).

Figure 2 and Figure 3 show the effect of blistering gas type, temperature and relative humidity on mechanical losses of the Nafion 211 and Nafion XL membrane, respectively. The results indicate that the elapsed time of mechanical loss is proportional to Van der Waals volume and radii of blistering gas type. As a result, hydrogen gas is the most effective blistering gas for inducing dynamic mechanical resistance loss in membranes. Furthermore, higher temperatures and lower relative humidity seem to cause more dynamic mechanical resistance loss. This is because the amorphous domain of the membrane possessing higher mechanical strength decreases with increasing temperature. It is speculated that more water molecules act as a good plasticizer at relatively higher humidity because the membranes gradually reduce their toughness and extend stress relaxation time [28]. It is also observed that the composite membrane Nafion XL (Figure 3) takes much longer than Nafion 211 (Figure 2) to reach 15% mechanical loss under the same blistering conditions, presumably due to the improved mechanical properties of Nafion XL, as shown in Table 1 [29].

### 3.2. Proton Conductivity Changes after Hydrogen Gas Blistering Aging

**Figure 4:**
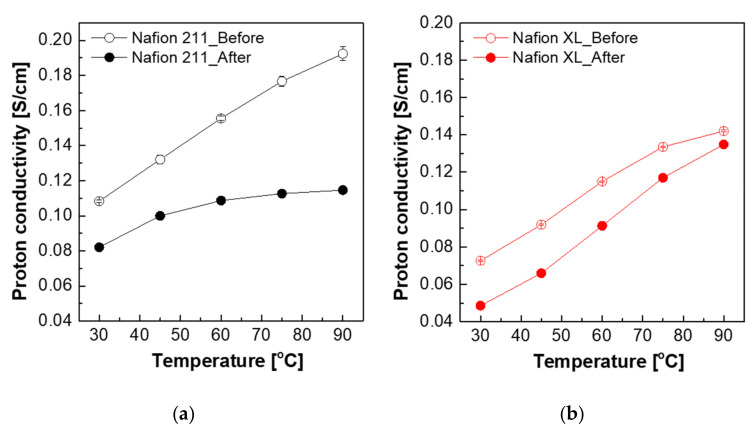
Proton conductivity of (**a**) Nafion 211 and (**b**) Nafion XL before and after pressure-loaded blistering.

**Figure 5:**
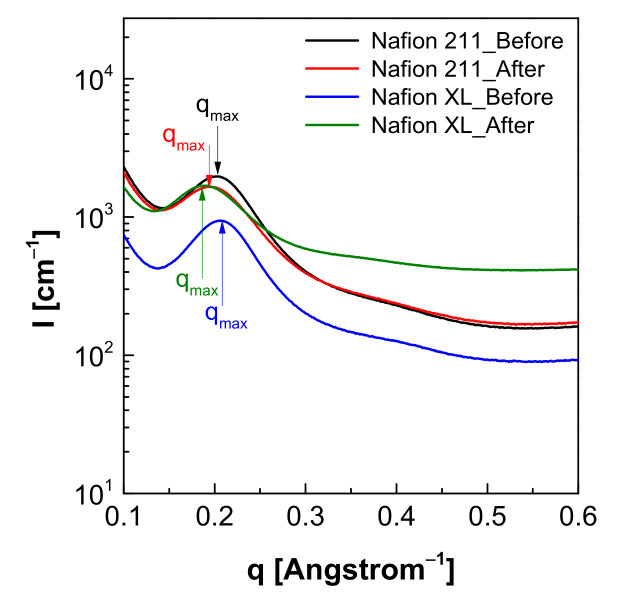
SAXS profile of Nafion 211 and Nafion XL membranes before and after pressure-loaded blistering.

Based on the above results, hydrogen gas was chosen as the blistering gas for the following tests. The changes of proton conductivity results before/after hydrogen gas blistering are presented in Figure 4. Before blistering, the proton conductivity of all the membranes rises with the temperature increased in the range of 30 °C to 90 °C. This temperature dependent proton conductivity has been also reported by other research groups [30]. The proton conductivity of all membranes sharply dropped after the pressure-loaded blistering, particularly for the non-reinforced membrane (Nafion 211), presumably due to the morphological changes such as the rearrangement or the deformation of the ionic channels [31]. In contrast to Nafion 211, the Nafion XL membrane has additional PTFE-support reinforcements and cerium-based radical scavengers to exhibit improved mechanical and chemical stability [32], which can be evidenced by SAXS results (Figure 5 and Table 2). From the SAXS analysis, the size of the hydrophilic domains for the ion passway grew longer after hydrogen gas blistering from 3.10 nm to 3.22 nm for Nafion 211, and from 3.03 nm to 3.31 for Nafion XL, respectively [33]. Consequently, this trend was also consistent with the results of proton conductivity changes [34].

### 3.3. Hydrogen Permeability Changes after Hydrogen Gas Blistering Aging

**Figure 6:**
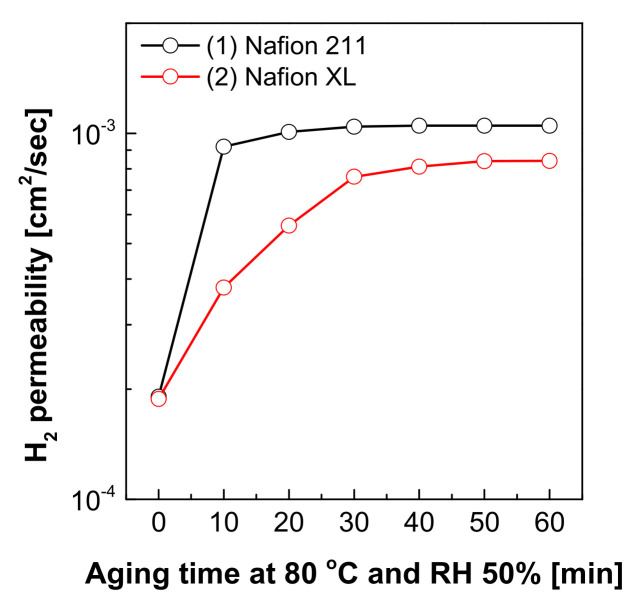
Gas permeability of Nafion 211 and Nafion XL membranes after dynamic mechanical aging using the PBS-GC system.

The hydrogen permeation could commonly indicate the degree of membrane mechanical durability. [35]. As shown in Figure 6, the membranes after dynamic mechanical aging (by pressure-loaded blistering time for 10 min) revealed rapid hydrogen permeability compared with the original membranes, which presumably suggested that the dynamic mechanical aging caused chemical degradation and microstructure mechanical damage of the membranes. The Nafion XL membrane showed a relatively higher mechanical stability compared to Nafion 211 in the change of hydrogen permeability after mechanical aging. Interestingly, increasing the blistering time (>10 min) does not induce more rapid hydrogen permeability of all the membranes. This observation indicates that the PBS-GC system based on the developed protocol is capable of screening and characterizing desirable membrane properties in a significantly reduced testing time.

## 4. Conclusions

Dynamic mechanical fatigue of PEMs is considered to be the main factor affecting the performance and life durability of PEMFCs for FCEVs. A relatively rapid approach has developed to determine the dynamic mechanical fatigue of PEMs by combining a pressure-loaded blister with a GC. The mechanical resistance of PEMs was tested at various temperatures, relative humidity, and applied pressure. The results show that the loss of mechanical resistance was faster as the applied temperature and load-pressure increased possibly due to the chemical and mechanical degradation of the membranes and high gas mobility. On the other hand, the loss of mechanical resistance was slower as the applied humidity increased due to the higher plasticization in the presence of more water molecules. It was also found that hydrogen among other applied gases is the most effective gas that notably influenced the loss of mechanical resistance because of its’ smallest Van der Waals volume and radius. Upon hydrogen blistering at 80 °C and 50% relative humidity, the proton conductivity of the membranes dropped remarkably. This result confirmed that there is a correlation with the interdomain distance obtained through SAXS analysis.

Finally, hydrogen permeation rate in the PEMs sharply increased with the pressure-loaded blistering for 10 min, but there were no significant changes after a certain minutes. This observation strongly indicated that the mechanical fatigue mainly occurred in the early stage of blistering time. As such, our developed approach can readily serve as a novel and rapid method to validate the durability of PEM materials for various applications including PEMFCs for FCEVs.

## Figures and Tables

**Table 1:** Physical properties of membranes.

	Type	Thickness (μm)	Basic Weight (g/m^2^)	Tensile Strength Max (MPa)	Elongation to Break (%)
Nafion^TM^ 211	Non-reinforced membrane	25.4	50	23 (MD), 28 (TD)	252 (MD), 311 (TD)
Nafion^TM^ XL	Composite membrane	27.5	55	45 (MD), 40 (TD)	200 (MD), 185 (TD)

**Table 2:** Morphological information of the membranes based on their SAXS profile.

	Interdomain Distance ^α^ (nm)	*q*_max_ (Å^−1^)
Nafion 211_Before	3.10	0.202
Nafion 211_After	3.22	0.194
Nafion XL_Before	3.03	0.207
Nafion XL_After	3.31	0.189

^α^ Obtained via theoretical conversion using Bragg’s law (Equation (2)) with each *q*_max_ value.
